# The Protective Effects of Salidroside from Exhaustive Exercise-Induced Heart Injury by Enhancing the *PGC-1* ***α***–*NRF1/NRF2* Pathway and Mitochondrial Respiratory Function in Rats

**DOI:** 10.1155/2015/876825

**Published:** 2015-06-16

**Authors:** Zheng Ping, Long-fei Zhang, Yu-juan Cui, Yu-mei Chang, Cai-wu Jiang, Zhen-zhi Meng, Peng Xu, Hai-yan Liu, Dong-ying Wang, Xue-bin Cao

**Affiliations:** ^1^Department of Cardiology, Geriatric Cardiovascular Disease Research and Treatment Center, No. 252 Hospital of PLA, Baoding 071000, China; ^2^Institute of Chinese Materia Medica, Guangxi University of Chinese Medicine, Nanning 530200, China; ^3^School of Preclinical Medicine, Youjiang Medical University for Nationalities, Baise, Guangxi 533000, China

## Abstract

*Objective*. To test the hypothesis that salidroside (SAL) can protect heart from exhaustive exercise-induced injury by enhancing mitochondrial respiratory function and mitochondrial biogenesis key signaling pathway *PGC-1α–NRF1/NRF2* in rats. *Methods*. Male Sprague-Dawley rats were divided into 4 groups: sedentary (C), exhaustive exercise (EE), low-dose SAL (LS), and high-dose SAL (HS). After one-time exhaustive swimming exercise, we measured the changes in cardiomyocyte ultrastructure and cardiac marker enzymes and mitochondrial electron transport system (ETS) complexes activities *in situ*. We also measured mitochondrial biogenesis master regulator *PGC-1α* and its downstream transcription factors, *NRF1* and *NRF2*, expression at gene and protein levels. *Results*. Compared to C group, the EE group showed marked myocardium ultrastructure injury and decrease of mitochondrial respiratory function (*P* < 0.05) and protein levels of *PGC-1α*, *NRF1*, and *NRF2* (*P* < 0.05) but a significant increase of *PGC-1α*, *NRF1*, and *NRF2* genes levels (*P* < 0.05); compared to EE group, SAL ameliorated myocardium injury, increased mitochondrial respiratory function (*P* < 0.05), and elevated both gene and protein levels of *PGC-1α*, *NRF-1*, and *NRF-2*. *Conclusion*. Salidroside can protect the heart from exhaustive exercise-induced injury. It might act by improving myocardial mitochondrial respiratory function by stimulating the expression of *PGC-1α–NRF1/NRF2* pathway.

## 1. Introduction

Exercise training is a double-edged sword, as proper intensity exercise is beneficial to human health, whereas excessive exercise can do harm to the body, especially the heart. Heart injury induced by excessive exercise training includes severe arrhythmia, heart failure, and even sudden cardiac death, which are common in military and athletic training. Research on heart injury induced by exhaustive exercise is quite important, yet few systematic studies of heart injury induced by exhaustive exercise have been published and the underlying signaling pathway mechanisms remain to be elucidated.

The term “heart failure energy starvation” was proposed decades ago [1]; however, very little is currently known about the origins of energetic failure. It appears that the transcriptional coactivator peroxisome proliferator-activated receptor-*γ* coactivator-1*α* (*PGC-1α*), which is a master regulator of mitochondrial biogenesis [2], plays a role in controlling the rate of the mitochondrial proliferation. Energy deficit and* PGC-1α* are markers of heart dysfunction, which can lead to impaired energy metabolism and contribute to heart failure. The term mitochondrial biogenesis refers to mitochondrial proliferation. Exercise, cold, energy restriction, oxidative stress, and other environmental stresses can all induce mitochondrial biogenesis. The discovery that maladapitive accumulation of mitochondrial biogenesis can lead to pathological phenomena, such as myocardial hypertrophy and heart failure, highlights the importance of elucidating the molecular regulatory mechanism for mitochondrial biogenesis.

Mitochondrial biogenesis requires the coordinated expression of the nuclear and mitochondrial DNA; the signaling pathways that coordinate the transcription and replication signaling pathways between genomic and mitochondrial DNA remain to be fully elucidated. It has been reported that* PGC-1α* plays a key role in skeletal muscle mitochondrial biogenesis and its expression level is rate limiting for skeletal muscle mitochondrial gene expression.* PGC-1α* cooperates with nuclear respiratory factors (*NRFs*), including* NRF1* and* NRF2*, and promotes the expression of multiple nuclear-encoded genes and the mitochondrial transcriptional factor A (*Tfam*). Nevertheless, some points of debate remain; for example, the expression of* PGC-1α* mRNA did not change after acute or endurance exercise training and the gene and protein levels of* NRF1 *and* NRF2 *were not correlated with* PGC-1α* gene or protein content [3].

Enzymatic assays for individual mitochondrial respiratory chain complexes have been widely used to estimate mitochondrial function and dysfunction. However, it has been established that this approach is not sufficient for a complete analysis of a potential mitochondrial injury, as it cannot reveal interactions between enzyme complexes. Additionally, routine mitochondrial isolation procedures will result in altered mitochondrial morphology and damaged function [4]. Respirometry [5] offers a powerful and physiologically relevant method to characterize coupled respiratory function in permeabilized tissue. A specially designed substrate-inhibitor titration approach allows for the step-by-step analysis of several mitochondrial complexes. However, the specific adaptive changes of myocardial mitochondrial respiratory function after exhaustive exercise remain unclear.

The stems of* Rhodiola crenulata* have been used as a traditional Chinese medicine for more than 1000 years [6].* R. crenulata* has the effect of supplementing qi (vital energy) and activating blood circulation and has been recognized as a plant-derived adaptogen that is capable of maintaining physiological homeostasis upon exposure to stress. Salidroside (SAL) is an effective extract component from* R. crenulata*. Many studies have found that SAL had protective effects on myocardial ischemia reperfusion [7], myocardial hypoxia [8], and myocardial injury [9]. Our previous research has suggested that SAL could improve hypoxia-induced cardiac myocyte energy metabolism by increasing the intracellular activity of the respiratory enzyme succinate dehydrogenase (SDH) [10]. However, there is no research regarding whether SAL is protective against acute myocardial injury caused by exhaustive exercise at present or not. In this study, we established the model of exhaustive swimming exercise-induced rat heart injury to investigate the effect of SAL on cardiac mitochondrial respiratory function and discuss whether the protection mechanism is through mitochondrial biogenesis* PGC-1α *–*NRF1*/*NRF2* signaling pathway or not.

## 2. Methods

### 2.1. Materials

SAL (98%) was purchased from Nanjing Zelang Medical Technology Co. (Lot: ZL201204012A) (Nanjing, Jiangsu, China). ELISA kits for heart injury markers were obtained from BD (New York City, NY, USA). Reagents for measuring mitochondrial respiratory function were purchased from Sigma-Aldrich (St. Louis, MO, USA). Antibodies to* PGC-1α*,* NRF1*,* NRF2*, and *β*-actin protein were purchased from CST (USA).

We obtained 40 male Sprague-Dawley rats (190–200 g) from Laboratory Animal Center of the Academy of Military Medical Sciences (Beijing, China), certification number SCXK: 2003-1-003. Animals were housed at 25°C-26°C under a 12 h dark, 12 h light cycle. Food and water were provided* ad libitum* both prior to and during the 1-day experimental period. All procedures were performed in accordance with the guidelines established by* the Convention for the Protection of Laboratory Animals of the PLA 252nd Hospital*.

A high-resolution respirometry (Oroboros Instruments, Austria), a microplate reader (Thermo Fisher SC, FI), a biochemical analyzer (7600-02, HITACHI, Japan), and a transmission electron microscope (TEM) (H-7500, HITACHI, Japan) were used in the experiments.

### 2.2. Experimental Groups and Exercise Training

Rats were trained in a water sink for 3 days prior to the formal experiment and those rats that were unable to adapt to swimming were excluded. A total of 32 rats ultimately entered the formal experiment. They were randomly divided into 4 groups: the sedentary control group (C, *n* = 8), the exhaustive exercise group (EE, *n* = 8), the pretreatment with low-dose SAL group (LS, *n* = 8), and the pretreatment with high-dose SAL group (HS, *n* = 8). The C and EE groups were administered 0.9% NaCl (12 mg·kg^−1^·d^−1^) intragastrically for 14 days and the LS and HS groups were administered 100 mg·kg^−1^·d^−1^ or 300 mg·kg^−1^·d^−1^ SAL, respectively, intragastrically for 14 days [11].

Rats in the EE, HS, and LS groups were subjected to one-time exhaustive swimming tests individually in a water sink (60 cm × 90 cm × 50 cm), with a water depth of 50 cm and temperature of 35°C ± 0.5°C. Exhaustion was defined by two criteria: greater than 10 s spent below the surface and lack of a “righting reflex” when placed on a flat surface [12]. At the point of exhaustion, all rats were anesthetized by the intraperitoneal injection of pentobarbital (50 mg·kg^−1^). Serum was collected and preserved at −80°C and the hearts were removed. The left ventricular tissue was isolated and part was immediately used for mitochondrial function measurements and part was preserved at −80°C for further analyses.

### 2.3. Histomorphometric Analysis

Rat hearts were fixed in 10% formaldehyde, preserved at room temperature, and observed using an Optical Microscope after H&E staining.

A small piece (2 mm × 1 mm × 1 mm) of subendocardial myocardium from the root of the left ventricular papillary muscle was harvested and fixed in 0.1 mmol/L phosphate buffer and observed by transmission electron microscopy (TEM).

### 2.4. Assay for Measuring Cardiac Marker Enzyme Activities

Enzyme linked immunosorbent assay (ELISA) kits were used to determine the levels of mitogen-activated protein kinases (CK), creatine kinase isoenzyme (CK-MB), lactate dehydrogenase (LDH), myoglobin (MB), and troponin (cTn-I) in serum. All assays were performed according to the manufacturer's instructions.

### 2.5. *In Situ* Studies of Mitochondrial Respiratory Function

We used permeabilized myocardial fibers, which provide an excellent way to study the mitochondria* in situ* without isolating them from tissue. Myocardial fibers were isolated by dissecting muscle tissue (left ventricle) in BIOPS solution on ice followed by saponin permeabilization. Cell membrane permeabilization with saponin enables the study of organelle function while maintaining cellular architecture and controlling the intracellular milieu. Mitochondrial function was measured by high-resolution respirometry at 37°C using dual-chamber titration injection respirometers. The respiration medium (MiR05) included 110 mM sucrose, 60 mM K-lactobionate, 0.5 mM EGTA, 1 g/L bovine serum albumin (essentially fatty acid-free), 3 mM MgCl_2_, 20 mM taurine, 10 mM KH_2_PO_4_, and 20 mM HEPES (pH 7.1). DatLab software (Oroboros Instruments) was used for data acquisition and analysis. A series of respiratory titration protocols were designed to test for multiple mitochondrial defects, including cytochrome c depletion. We used 1 mM adenosine diphosphate (ADP) to stimulate respiration (state 3) and measured it sequentially through complex I (10 mM glutamate and 2 mM malate), complex II (10 mM succinate and 0.5 *μ*M rotenone), and complex IV (0.5 mM TMPD, 5 mM ascorbate, and 2.5 *μ*M antimycin A). Chemical background controls were used to correct for TMPD autoxidation and ascorbate. Respiration was measured before and after stimulation by adding cytochrome c (10 *μ*M). Respiratory rates were expressed per mg dry weight and measured on samples collected from the oxygraph chamber at the end of the experiment.

### 2.6. Real-Time Polymerase Chain Reaction

RNA was isolated from frozen rat myocardia using an ultrapure RNA kit according to manufacturer's protocol. Real-time PCR was performed and analyzed using a fluorescent PCR instrument (IQ-5) using cDNA and SYBR Green PCR Master Mix. Primers sequences are listed in Table 1. The relative amounts of mRNA were determined based on 2^−ΔΔCt^ calculations.

### 2.7. Western Blotting

Tissue protein reagent was used to extract protein. The protein concentration was determined using the bicinchoninic acid assay (BCA). Then, the protein was added to SDS-PAGE sample loading buffer after dilution in a similar volume and it was placed in a 100°C water bath for 5 min. Protein samples were separated by SDS-PAGE at 100 V for 15 min and 150 V for 45 min and were then transferred to polyvinylidene fluoride (PVDF) membranes. Membranes were blocked in 5% skim milk blocking buffer at room temperature for 1.5 h and then were incubated overnight at 4°C with primary antibodies. After washing the samples with Tris-buffered saline (TBS) Tween, the membranes were incubated with secondary antibody for 2 h at room temperature. ECL was used for colorimetric detection for 5 min after washing with TBS Tween three times. Values were normalized to those of the internal control (*β*-actin).

### 2.8. Statistical Analyses

SPSS version 16.0 (SPSS Inc., Chicago, IL, USA) was used for statistical analyses. Results were expressed as means ± SD. *t*-test was used to compare data between two groups. One-way analysis of variance (ANOVA) was used to compare data among multiple groups. Statistical significance was considered when *P* < 0.05.

## 3. Results

### 3.1. The Effect of Exhaustive Exercise and Salidroside Interference on Histomorphometric Analyses

#### 3.1.1. Light Microscopy

**Figure 1:**
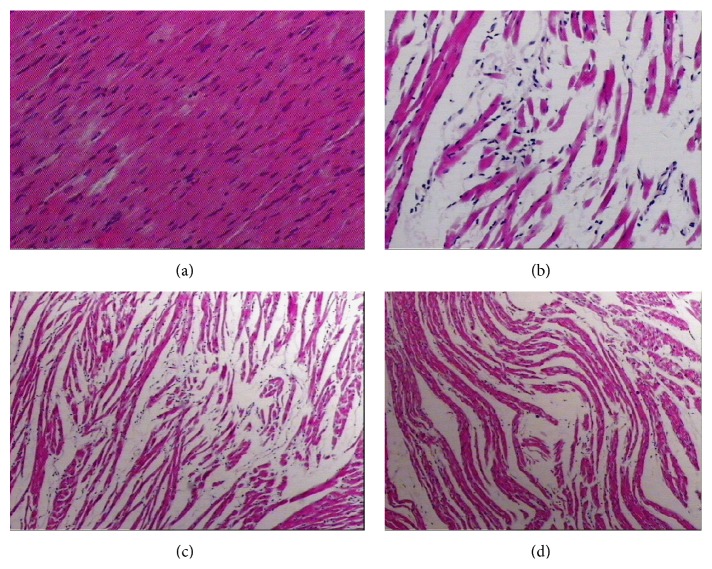
The effect of exhaustive exercise and SAL on the myocardial morphology. Hematoxylin and eosin (H&E) staining (×400); (a) the C group, (b) the EE group, (c) the LS group, and (d) the HS group.

Figure 1 showed an optical microscopy analysis of the rat myocardial structure.  Figure 1(a) shows that, in the C group, muscle fibers were arranged neatly as an interstitial substance without edema, the muscle membranes showed no damage, and the muscle fibers had no fractures, degeneration, or necrosis.  Figure 1(b) showed that the EE group myocardial fibers were arranged irregularly as interstitial substance with edema, there was muscle membrane damage, and the muscle fibers showed evidence of fracture, degeneration, and necrosis. Figures 1(c) and 1(d) showed that, in the LS and HS groups, the muscle fiber direction changed, the interstitial areas showed slight edema, and the muscle membrane had no damage.

#### 3.1.2. Electron Microscopy

**Figure 2:**
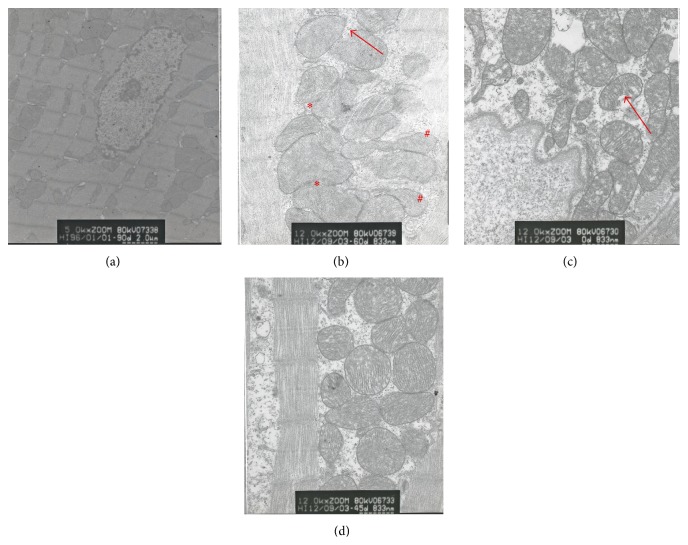
The effect of SAL on cardiomyocyte ultrastructure (×20,000); (a) the C group, (b) the EE group, (c) the LS group, and (d) the HS group. Note the disrupted mitochondrial membrane (arrows), mitochondrial swelling and fusion (*∗*), and mitochondrial malformation (#).

Figure 2 showed the cardiomyocyte ultrastructure.  Figure 2(a) showed a TEM structure representative of C group rats: sarcomeres were arranged neatly, the density was uniform, the organelles had no edema, and the membrane and crest of the mitochondria were normal.  Figure 2(b) showed the myocardial structures of the EE group rats: the myocardial nuclear matrix had edema, the nuclear gap widened, the number of mitochondria and glycogen content decreased significantly, the membrane and crest of the mitochondria partially fused and became blurry or missing, and a small amount of muscle fiber was necrotic. Figures 2(c) and 2(d) showed the myocardial structure of the LS and HS group rats, which was as follows: the myocardial cell matrix had edema and the membrane and mitochondrial crest partially fused and became blurry or absent.

### 3.2. The Effect of EE and SAL Interference on Enzyme Markers of Heart Injury

As shown in Table 2, compared to the C group, LDH, CK-MB, CK, CTN-I, and MB in the EE group increased significantly (*P* < 0.05); compared to the EE group, LDH, CK-MB, CK, CTN-I, and MB of the LS group were reduced significantly (*P* < 0.05); and compared to the LS group, LDH, CK-MB, CK, and MB of the HS group were also reduced significantly (*P* < 0.05).

### 3.3. The Effect of EE and SAL Interference on Mitochondrial Respiration Function

**Figure 3:**
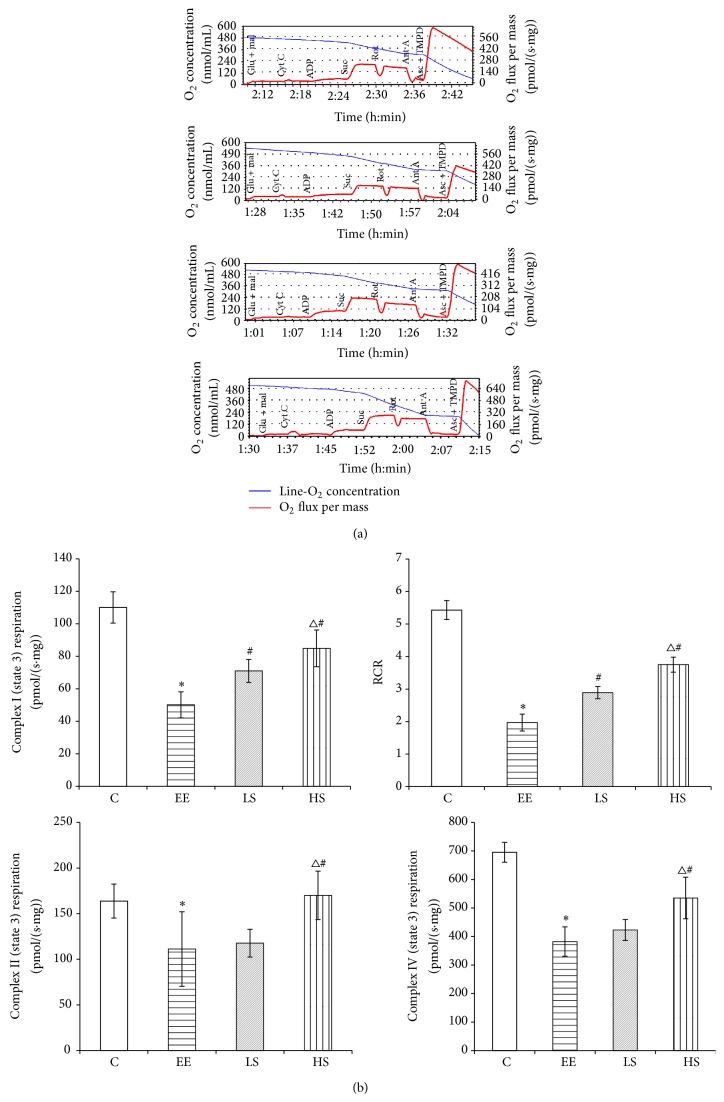
Maximal respiratory capacity (state 3 respiration) in permeabilized myocardial fibers. (a) Original recording. (b) Comparison. Glu, glutamate; Mal, malate; Cyt C, cytochrome C; Suc, succinate; Rot, rotenone; Ant A, antimycin A; and Asc, ascorbate. Data were expressed as means ± SD; *n* = 8 per group; ^*∗*^ *P* < 0.05 versus the C group; ^#^ *P* < 0.05 versus the EE group; and ^Δ^ *P* < 0.05 versus the LS group.

As illustrated in Figure 3, titration of cytochrome c did not alter the flux, indicating that the mitochondrial outer membrane was intact. With glutamate and malate as electron donors for complex I, compared to the C group, the state 3 respiration rate of the EE group was reduced significantly (*P* < 0.05); compared to the EE group, the LS was increased significantly (*P* < 0.05); and compared to the LS group, HS was increased significantly (*P* < 0.05). Compared to the C group, the RCR of the EE group was reduced significantly (*P* < 0.05); compared to the EE group, the RCR of LS and HS groups were both increased significantly (*P* < 0.05).

Using succinate as a substrate for complex II, compared to the C group, the state 3 respiration rate of the EE group was reduced significantly (*P* < 0.05); compared to the EE group, the LS was increased insignificantly (*P* > 0.05) and the HS was increased significantly (*P* < 0.05); and compared to the LS groups, the HS was increased significantly (*P* < 0.05).

With ascorbate/TMPD being used as substrates for complex IV, compared to the C group, the state 3 respiratory rate of the EE group was reduced significantly (*P* < 0.05); compared to the EE group, the LS was increased insignificantly (*P* > 0.05) and the HS was increased significantly (*P* < 0.05); and compared to the LS group, the HS was increased significantly (*P* < 0.05).

### 3.4. The Effects of EE and SAL Interference on* PGC-1α*,* NRF1*, and* NRF2* Gene Expression Levels

**Figure 4:**
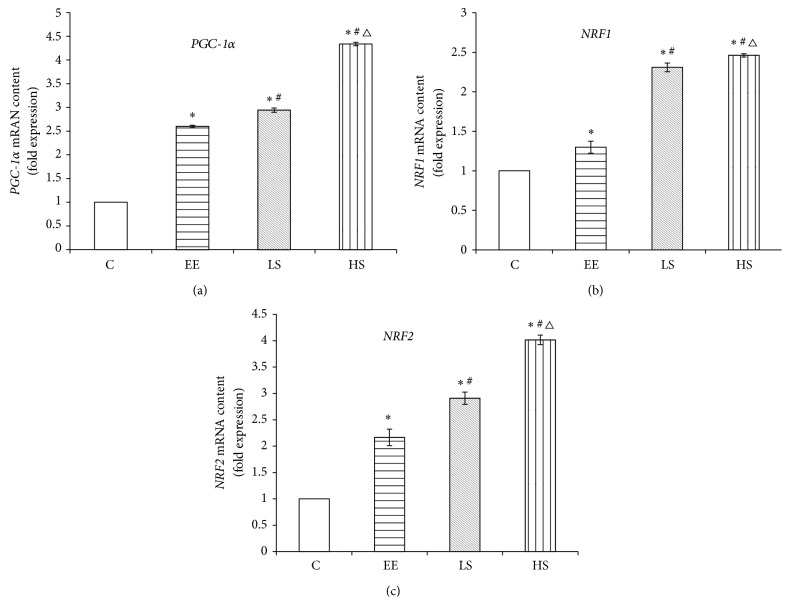
*PGC-1α*,* NRF1*, and* NRF2* mRNA expression levels in different groups. Data were expressed as means ± SD; *n* = 8 per group; ^*∗*^ *P* < 0.05 versus the C group; ^#^ *P* < 0.05 versus the EE group; and ^Δ^ *P* < 0.05 versus the LS group.

As shown in Figure 4, compared to the C group,* PGC-1α*,* NRF1*, and* NRF2* mRNA expression levels in the EE group were all significantly elevated (*P* < 0.05); compared to the EE group,* PGC-1α*,* NRF1*, and* NRF2 *mRNA expression levels in the LS group were elevated significantly (*P* < 0.05); and compared to the LS group,* PGC-1α*,* NRF1*,and* NRF2* mRNA expression levels in HS group were all significantly elevated (*P* < 0.05).

### 3.5. The Effect of Exhaustive Exercise and Salidroside Interference on* PGC-1α*,* NRF1*, and* NRF2* Protein Expression

**Figure 5:**
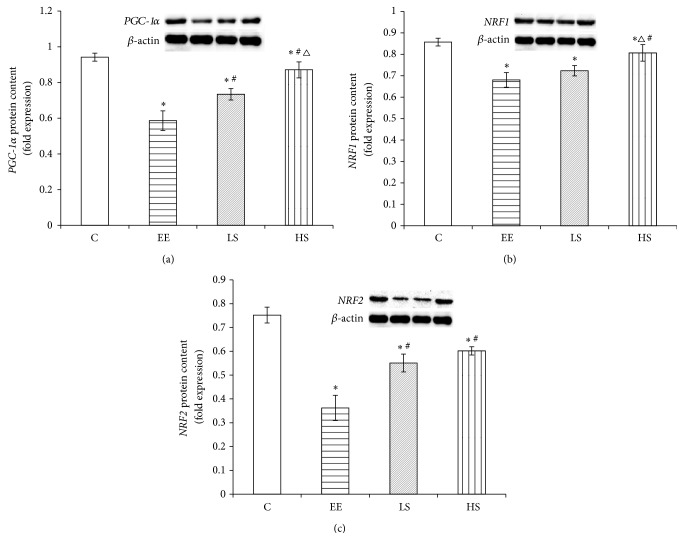
*PGC-1α, NRF1*, and* NRF2* protein expression levels in different groups. Data were expressed as means ± SD; *n* = 8 per group; ^*∗*^ *P* < 0.05 versus the C group; ^#^ *P* < 0.05 versus the EE group; and ^Δ^ *P* < 0.05 versus the LS group.

As shown in Figure 5, compared to the C group,* PGC-1α*,* NRF1*, and* NRF2* protein levels in the EE group were reduced significantly (*P* < 0.05); compared to the EE group,* PGC-1α*,* NRF1*, and* NRF2* protein levels in the LS group were significantly elevated (*P* < 0.05); and compared to the LS group,* PGC-1α*,* NRF1*, and* NRF2* protein levels in the HS group were increased significantly (*P* < 0.05).

## 4. Discussion

In this study, we investigated the protective effect and underlying mechanism of SAL on exhaustive exercise-induced heart injury with a focus on energy metabolism. We mainly observed alterations of myocardial structure, myocardial injury enzyme markers, and mitochondrial respiratory function in permeabilized fibers in response to exhaustive exercise. We also observed the expression of the key mitochondrial biogenesis signaling pathway* PGC-1α*–*NRF1*/*NRF2* to characterize the cellular and molecular mechanism of exhaustive exercise-induced heart injury.

The serum marker analyses suggest that CK, CK-MB, LDH, cTn-I, and MB were released into blood, so the contents of these enzymes in the serum increased significantly, indicating that the cardiomyocytes were injured. Applying morphometric analysis to optical and electron microscopy specimens, we found that the characteristics of the EE group rat myocardia were nuclear matrix edema, nuclear gap widening, an increased number of mitochondria and amount of glycogen, partial membrane and mitochondrial crest fusion that became blurry or absent, and a small amount of muscle fiber necrosis. Those findings indicate that cardiomyocyte structure was damaged as a consequence of exhaustive exercise.

Muscle oxidative capacities have been assessed by measuring respiration of mitochondria in permeabilized fibres with no limitation of substrates, ADP, or oxygen [13]. Results showed that the state 3 of ETS complexes I, II, and IV was decreased 55%, 32%, and 45%, respectively. This suggests that exhaustive exercise induced depression of myocardial mitochondrial respiration function. In order to fulfill the energy needs of myocytes, the ETS would compensate with producing large amount of superoxide which in turn injures myocardium [14–16] and causes the respiration apparatus damage and energy production failure. In contrast, the treated group showed improvements for the above indicators, particularly for the high SAL dose group that had clearly showed improved energy metabolism. These data indicate that SAL can protect from exhaustive exercise-induced heart injury by improving mitochondrial respiratory function and energy metabolism.

The coactivator* PGC-1α* plays a central role in a regulatory network that governs the transcriptional control of mitochondrial biogenesis and respiratory function. Through its interaction with multiple transcription factors,* PGC-1α* enhances mitochondrial capacity for oxidative phosphorylation and triggers the coordinate expression of nuclear- and mitochondrial-encoded genes driving mitochondrial biogenesis [17].

Actually, two distinct classes of regulatory proteins govern mitochondrial biogenesis at the transcriptional level in mammalian systems. The first class comprises transcription factors mitochondrial transcription factor A (Tfam) and the mitochondrial transcription factor B (mtTFB) isoforms TFB1M and TFB2M, which direct transcription from divergent heavy- and light-strand promoters within the mitochondrial D-loop regulatory region. A second group of transcription factors act on the majority of nuclear genes whose products are required for respiratory chain expression and biological function. Among these are the nuclear respiratory factors* NRF1* and* NRF2* [18]. Since we mainly observed the mitochondrial respiration function performance in this research, we attached more importance to* NRF1* and* NRF2*.

In this research, expression levels of* PGC-1α*,* NRF1*, and* NRF2* mRNA were upregulated compared to the control groups immediately after the exercise; however,* PGC-1α*,* NRF1*, and* NRF2* proteins were reduced 1.6-fold, 14.6%, and 52%, respectively. This inconsistency between mRNA and protein expression levels might indicate that exhaustive exercise caused mitochondrial biogenesis protein synthesis or assembly processes dysfunction. The downregulation of* NRF1* and* NRF2 *proteins is also involved in dysfunctional synthesis of mitochondrial respiration complex subunits, resulting in reduced mitochondrial respiration function [19, 20]. Therefore, decreased* PGC-1α* and its downstream transcription factors* NRFs* proteins contribute to deficiency in myocardial oxidative capacity and energy production [1]. These results indicate that exhaustive exercise is such an intense stress that it damages mitochondrial respiration function which is responsible for energy production by downregulating the mitochondrial biogenesis key regulatory factors* PGC-1α*–*NRF1*/*NRF2* signaling pathway. The duration of the stress will in turn worsen the imbalance between energy production and energy demand to satisfy the contractile function of cardiac muscle, which will lead to irreversible myocardial damage and energy failure. In contrast, the SAL treated group particularly the high-dose group significantly upregulated* PGC-1α*,* NRF1*, and* NRF2*, indicating that SAL can improve energy metabolism by upregulating mitochondrial biogenesis regulators and ameliorate myocardial injury induced by intense exercise.

To date, studies have examined changes in* PGC-1α* in a variety of exercise models of heart. Botta et al. [3] reported that short-term and moderate-intensity exercise upregulated* PGC-1α*. A five-week high-intensity exercise training regimen resulted in a 41.5% increase in the* PGC-1α* mRNA expression levels [21]. Those controversial results probably vary depending on exercise intensity.

In this study, SAL significantly reduces the levels of myocardial injury enzymes in the serum, relieves the myocardium ultrastructure injury, improves the activity of mitochondrial ETS complexes I, II, and IV, and increases expression of key mitochondrial biogenesis factors* PGC-1α*,* NRF1*, and* NRF2*. All of these findings indicate that SAL can protect the heart from exhaustive exercise-induced injury by improving mitochondrial respiratory function and mitochondrial biogenesis.

In conclusion, exhaustive exercise can induce heart injury, including structural injury, enzyme abnormalities, reduced respiratory function, and the downregulation of mitochondrial biogenesis. The accumulation of these maladaptive traits initiates a vicious cycle that can further cause myocardial metabolic dysfunction, thereby contributing to myocardial injury and heart failure. SAL, one of the major effective components of extracts from the traditional Chinese medicine* R. crenulata*, appears to protect the myocardium from exhaustive exercise-induced injury by improving respiratory function and upregulating mitochondrial biogenesis.

## Figures and Tables

**Table 1:** Primers used for real-time PCR analyses of mRNA expression.

	Forward	Reverse
*PGC-1α*	5′-ACCCACAGGATCAGAACAAACC-3′	5′-GACAAATGCTCTTTGCTTTATTGC-3′
*NRF1 *	5′-GGCACAGGCTGAGCTGATG-3′	5′-CTAGTTCCAGGTCAGCCACCTTT-3′
*NRF2 *	5′-CCTAAAGCACAGCCAACACA-3′	5′-ACAGTTCTGAGCGGCAACTT-3′
*β*-actin	5′-GGCTGTATTCCCTCCATCG-3′	5′-CCAGTTGGTAACAATGCCATGT-3′

**Table 2:** A comparison of heart injury serum markers in different groups.

Groups	LDH	CK-MB	CK	CTN-I	MB
C	0.92 ± 0.04	2.11 ± 0.18	11.70 ± 0.46	144.61 ± 12.71	3.32 ± 0.49
EE	1.08 ± 0.09^*∗*^	3.23 ± 0.19^*∗*^	16.93 ± 0.41^*∗*^	172.00 ± 12.83^*∗*^	4.90 ± 0.41^*∗*^
LS	1.04 ± 0.06^#^	3.14 ± 0.24	14.56 ± 0.53^#^	157.18 ± 16.95	4.22 ± 0.43^#^
HS	1.02 ± 0.10^#^	2.57 ± 0.21^#Δ^	13.19 ± 1.91^#Δ^	159.26 ± 8.93	3.98 ± 0.54^#Δ^

Data are expressed as means ± SD; *n* = 8 for each group; ^*^ *P* < 0.05 versus the C group, ^#^ *P* < 0.05 versus the EE group, and ^Δ^ *P* < 0.05 versus the LS group.
